# How to Be Eloquent

**Published:** 1855-06

**Authors:** 


### HOW TO BE ELOQUENT.

*Eloquence consists in feeling a truth yourself, and in making those who hear you, feel it.* Oratory is not vociferation ; it is not stamping a hole in the platform, nor beating all the dust out of the cushion of the pulpit ; nor tearing off your coat-tail in the violence of your gesticulations, a la Gavazzi ; it is not holding the breath until the face is purple and the eyes blood-shot ; it is not hissing through the teeth like the fizzle of a squib, nor crouching down, then bounding upward like a wild cat springing on a 'possum ; nor ranting about from one side of the rostrum to another until the skin is drenched in perspiration, and the body weakened into helplessness : you are not eloquent in all this, unless it be for the grave, for it is suicidal. I will tell you how :—The minister of my youth preached thus and perished—went from health to the grave in twenty-four hours. He was an earnest speaker in a small church, in cold weather at night from home ; he was invited to the house of an elder near by ; it was late, and they ushered him into their best room, seldom used except by the most favored ; no fire was kindled, the sheets were cold and damp. Being modest he said nothing, but hoped no harm would result from it ; but harm did result—the perspiration was checked at once—the efforts of the day had produced such physical exhaustion, that there was no power of reaction ; the result was a congestive chill, terminating fatally in a few hours. Not long since, a gentleman came to me, who had been an effective political speaker ; after the close of an exciting address, he rode several miles in a cold east wind, checking perspiration, falling on the throat, travelling downward to the lungs, and he will not live sixty days. The lives of ministers, oftener than those of any other class of persons, are thrown away in this manner constantly. The February number for last year advises as to conduct under such circumstances. I, as a world's wanderer, found out a long time ago, *that very often, the best side of a bed was the outside.* If the minister just referred to had lain on the bed with his clothes on, and drawn the cover over him, he might perhaps have rumpled his shirt collar or his dickey-bosom; but then he would, humanly speaking, have been alive to this day, working for the Master. It is wonderful, what multitudes of men blunder along life's pathway in utter thoughtlessness! This excellent gentleman was a fine scholar, and a leader of his brethren; he was our neighbor, and came in one day to request a younger brother to come and stop a hole in his fence to keep the pigs out from the street; he said he had been thinking how to do it himself, but could not make it out. He reminded me of another, some say it was Sir Isaac Newton, who had a favorite cat, and she had a parcel of kittens; as the weather was cold, he concluded to have a hole cut in a side door to enable her to come in and out; when that was done, it occurred to him that there was greater need for the little kittens to come in and get a warm once in a while, so he had a small hole for their accommodation cut beside the large one. And so it is with many learned men; they will not think about what they deem small matters, and in consequence often lose life, and with it all things. Let all remember, then, that perspiration suddenly checked in the way just named, or in any other, slays annually its thousands. Not even an "*iron constitution*" can bear with impunity suddenly checked perspiration.

Eloquence, as I was saying at the commencement of this article, is not in the tempest or the tornado; not in the rolling of the thunder nor in the coruscation of the fierce lightning, for inspiration says, it is in the *still small voice*, in the murmurings of the gentlest zephyr. So it is with man among his fellows; it is not boisterous wordyism, but calm and earnest truthfulness. We need more of heart and less of voice, if we would carry men with us, and take them captive agaist their will. In the stern, hard, dry place of a court-room, tears spring unbidden to eyes that seldom weep, as the judge passes sentence of long years of imprisonment or a terrible death; but whoever heard of a judge being boisterous there? And is not every sermon a "*savor*" of endless death to some? Be assured, our clergy must feel more; until then, the preaching of the gospel will be still, for the most part, as sounding brass, or a tinkling cymbal.

